# CDC’s Laboratory Activities to Support Newborn Screening for Spinal Muscular Atrophy

**DOI:** 10.3390/ijns10030051

**Published:** 2024-07-17

**Authors:** Francis K. Lee, Christopher Greene, Kristina Mercer, Jennifer Taylor, Golriz Yazdanpanah, Robert Vogt, Rachel Lee, Carla Cuthbert, Suzanne Cordovado

**Affiliations:** Newborn Screening and Molecular Biology Branch, U.S. Centers for Disease Control and Prevention, Atlanta, GA 30341, USA; crg0@cdc.gov (C.G.); kbutzemercer@gmail.com (K.M.); jtaylor@fnih.org (J.T.); czu1@cdc.gov (G.Y.); rfv1@cdc.gov (R.V.); nmo1@cdc.gov (R.L.); ijz6@cdc.gov (C.C.); snc4@cdc.gov (S.C.)

**Keywords:** newborn screening, spinal muscular atrophy, real-time PCR, quality assurance, proficiency testing, quality control, locked nucleic acid

## Abstract

Spinal muscular atrophy (SMA) was added to the HHS Secretary’s Recommended Uniform Screening Panel for newborn screening (NBS) in 2018, enabling early diagnosis and treatment of impacted infants to prevent irreversible motor neuron damage. In anticipation of supporting SMA newborn screening, scientists at the U.S. Centers for Disease Control and Prevention (CDC) have worked towards building resources for public health laboratories in four phases since 2013. In Phase 1, CDC established a real-time PCR assay, which uses a locked nucleic acid probe to attain the needed specificity, to detect *SMN1* exon 7. In Phase 2, we developed quality assurance dried blood spot materials made with transduced lymphoblast cell lines established from de-identified SMA patients, carriers, and unaffected donors. In 2021, CDC implemented Phase 3, a proficiency testing program, that now supports 115 NBS labs around the world. We are currently completing Phase 4, which includes the implementation of an external SMA quality control material program. Also, during this time, CDC has provided individual technical assistance to NBS programs and bench training to NBS scientists during our annual molecular workshop. These CDC-led activities have contributed to the rapid and full implementation of SMA screening in all 50 U.S. states as of February 2024.

## 1. Introduction

Spinal muscular atrophy (SMA) is the leading cause of infant mortality among heritable autosomal recessive disorders [1]. With a pan-ethnic prevalence of 1 in 10,000 to 1 in 12,000 births and a carrier frequency of 1/40–1/60, SMA is second in birth prevalence only to cystic fibrosis [2]. The disease is characterized by degeneration of alpha motor neurons in the spinal cord. Over time, SMA results in progressive and symmetrical proximal weakness, with key signs including floppy baby syndrome, hypotonia, and areflexia, leading to paralysis and ultimately premature death if untreated. SMA is phenotypically classified into three types based on age of onset and severity [1]. Symptoms of SMA Type I, the most common type, begin from birth to six months of age. These children are never able to sit unsupported and typically live for only two years due to respiratory complications. Children with Type II SMA start showing signs of the disease before the age of 18 months and have a life expectancy of less than 4 years. They can sit unassisted but never achieve the ability to walk. Children with the least severe type of childhood SMA, Type III, do not develop symptoms until after 18 months of age, are able to walk, and can live into adulthood [3].

As of 2024, the U.S. Food and Drug Administration (FDA) has approved three treatments that have been proven effective to alter the progression of SMA [4]. Spinraza^TM^, an antisense oligonucleotide that increases the production of survival motor neuron protein, which leads to significant improvement in achieving motor milestones among all three types of SMA patients, was approved in 2016. This treatment requires intrathecal injection at repeated intervals [5]. A functionally similar drug, Evrysdi^TM^, was approved by FDA in 2020 and allows non-invasive, daily at-home oral treatment [6]. Both drugs require lifelong administration. In 2019, FDA approved the use of the gene replacement therapy Zolgensma^TM^, which offers a one-time treatment option by replacing the function of the non-working *SMN1* gene [7]. However, none of these therapeutic modalities are a complete “cure” since spinal cord motor neurons have limited regeneration capacity, and mainly halt disease progression. Thus, detection of infants with SMA through newborn screening (NBS) provides an opportunity for early treatment to prevent postnatal motor neuron damage, including cases that are asymptomatic at birth. For these reasons, the U.S. Secretary of Health and Human Services added SMA to the Recommended Uniform Screening Panel (RUSP) in 2018 [8].

At the U.S. Centers for Disease Control and Prevention (CDC), we have monitored the development of SMA therapy since 2013 and have proactively engaged in laboratory activities to support SMA NBS in anticipation of a likely therapeutic breakthrough and the addition of SMA to the RUSP. This manuscript describes our efforts to facilitate the full implementation of SMA screening in all 50 U.S. states in February 2024 [9]. Our activities can be grouped into four sequential phases, each focused on a quality critical component to support the implementation of newborn screening in public health laboratories.

## 2. Materials and Methods

### 2.1. SMA NBS Assay Development

The CDC assay was designed to detect the most common pathogenic variant, a deletion and/or gene conversion of *SMN1* exon 7 using a real-time PCR assay platform. Since the specificity of a conventional *SMN1* real-time PCR assay is compromised by the presence of the *SMN2* gene, a paralog of the *SMN1* gene that is nearly identical in sequence, we incorporated locked nucleic acid (LNA) molecules in the Taqman^TM^ probe to exclude signals generated from the *SMN2* gene that may mask the absence of *SMN1* exon 7. An LNA is a modified RNA nucleotide in which the ribose moiety is modified with an extra bridge connecting the 2′ oxygen and 4′ carbon. This modification confines the ribose in the 3′-edo conformation, which increases its affinity for mismatch discrimination against non-complimentary nucleotides in binding. In our Taqman^TM^ probe, the LNA molecules were inserted at the C/T mismatched nucleotide location (c.840C>T; NM_000344.3) to increase duplex stability and improve specificity of hybridization between probe and target sequence at 60 °C [10]. The discriminating nucleotides were intentionally positioned in the center of the probe sequence following published recommendations for optimal LNA probe design [11]. Additional LNAs were incorporated at selected adjacent locations to increase the melting temperature (T_m_) of the probe and allow for more stringent cycling conditions (manuscript in preparation). The sequences of the primers and probe are as follows: forward primer, 5’-CTTGTGAAACAAAATGCTTTTTAACATCCAT-3’; reverse primer, 5’-GAATGTGAGCACCTTCCTTCTTTTT-3’; and probe, 5’-/5Cy5/GGTT+T+C+A+G+A+CAA/3IAbRQSp/-3’ (the + sign in front indicates an LNA nucleotide)

### 2.2. Dried Blood Spot (DBS) Quality Assurance (QA) Material Development

To produce cell-based DBSs for SMA QA, we first developed cell lines at CDC from SMA patients with a homozygous *SMN1* exon 7 deletion, from SMA carriers of an exon 7 deletion, and from donors with two normal exon 7 sequences. Blood samples were obtained from de-identified donors with appropriate consent through a CDC contract (75D30123C16559) with the Sequoia Foundation. The isolated peripheral blood mononucleated cells were transduced with Epstein–Barr virus to establish immortalized B lymphoblast cell lines [12]. The cell lines were grown in suspension cultures in spinner flasks to a high cell density (>1.0 × 10^6^/mL) to enable sustainable large-volume production. The cultured cells were harvested and added to leukocyte-depleted blood to a concentration of 15 million cells per mL. Seventy-five microliter aliquots of blood were spotted on to Whatman 903 filter paper cards (Whatman, Piscataway, NJ, USA) using the ViaFlow Assist robotic fluid handlers (Integra Biosciences, Hudson, NH, USA) to create DBSs.

### 2.3. Establishment of SMA Proficiency Testing Program

CDC launched the SMA proficiency testing (PT) program in 2020, first as a pilot program to U.S. and Canadian screening laboratories, and then transitioned it in 2021 to a routine PT program that is provided to domestic and international participants three times per year. Each PT panel contains five unknown DBSs that can be either an *SMN1* exon 7 deletion positive (i.e., homozygous specimens), an *SMN1* exon 7 deletion negative that includes 1 or 2 copies of *SMN1* exon 7 (i.e., carriers or *SMN1* normal specimens), or an unsatisfactory sample (i.e., both *SMN1* and the reference gene are out of range). PT participants are asked to test for the presence or absence of *SMN1* exon 7 and report their results to CDC, including the analytical method used, the DNA extraction method, and the internal reference gene used in the assay. PT results are analyzed at CDC, and a lab-specific performance report is provided to each participant, along with an aggregate summary report of all participants without identifiers.

### 2.4. Implementation of an SMA External Quality Control Program

To support the NBS community’s need for external quality control DBS materials, CDC launched a pilot external quality control (QC) program on 21 May 2024. After the pilot phase, the program will transition to a routine QC program that will be provided to participating newborn screening labs twice a year. Since many U.S. screening labs use a multiplexed assay that includes primers and probes that can detect biomarkers for both SMA and severe combined immunodeficiency (SCID), the QC program will include DBS QC materials for both disorders.

## 3. Results

### 3.1. Phase 1: Development of Real-Time PCR Newborn Screening Assay for SMA

Our assay was designed for newborn screening to identify infants affected with 5q13-linked SMA resulting from the homozygous loss of *SMN1* exon 7, which accounts for about 95% of all SMA cases. Most cases of 5q13-linked SMA are caused by a variant with complete deletion of the *SMN1* sequence or a partial 6.3-kilobase deletion that includes exon 7, intron 7, and exon 8 [13]. The paralog gene, *SMN2*, also encodes for the SMN protein; however, a single nucleotide difference between *SMN1* (C) and *SMN2* (T) located in exon 7 (c.840C>T; NM_000344.3) removes an exon splice enhancer site that results in an unstable transcript due to skipping of exon 7 during splicing of *SMN2* mRNA [14]. Initially, CDC developed an assay that targeted the *SMN1* intron 7 region using an LNA probe that showed excellent discrimination against *SMN2* intron 7 [15]. However, a subsequent publication from Taiwan investigators [16] using an assay based on the intron 7 target identified five false positive cases that had an *SMN1* exon 7–*SMN2* intron 7 hybrid allele. Because *SMN1* exon 7 was still present, these infants would not develop SMA, and a false positive case would result based on the absence of the *SMN1* intron sequence. Additionally, Chien et al. reported an allele frequency of 1:585 for the *SMN2* exon 7–*SMN1* intron 7 hybrid genotype, which could potentially result in a false negative screen using the intron-based assay. Based on these findings, we redesigned our assay using primers and a probe that specifically amplifies and detects *SMN1* exon 7, rather than the intron 7 region of the gene. Since there are only five mismatched nucleotides between *SMN1* and *SMN2* in this region, traditionally designed primers would amplify both genes. To attain the specificity for the *SMN1* exon 7 sequence, we again employed LNA nucleotides in the probe. We also evaluated if there was a specificity advantage to designing the probe to the forward (plus) strand compared to the reverse (minus) strand sequence. In the forward strand sequence of *SMN1*, we incorporated a “C” LNA (a pyrimidine) at the mismatch site, and in the reverse strand sequence, we incorporated a “G” LNA (a purine) at the mismatch site. As predicted by the difference in binding characteristics between the LNA pyrimidine and purine bases [10], the forward strand had higher specificity for *SMN1* than the reverse strand, as indicated by a lower non-specific fluorescent signal in SMA patient samples. To further evaluate the specificity, we challenged the reaction with a synthetic *SMN2* gene fragment that was used at an equivalent concentration of up to 40 copies per cell. No *SMN1* exon 7 signal was detected in all cases, further establishing the excellent specificity of the assay. We then successfully incorporated the *SMN1* exon 7 test reagents into the existing multiplex real-time PCR assay for T-cell receptor excision circle (TREC), a marker of T cell production, to detect severe combined immunodeficiency (SCID), which also included the target *RPP30* as an internal control for both assays (manuscript in preparation).

### 3.2. Phase 2: Development of Quality Assurance DBS Materials

**Figure 1:**
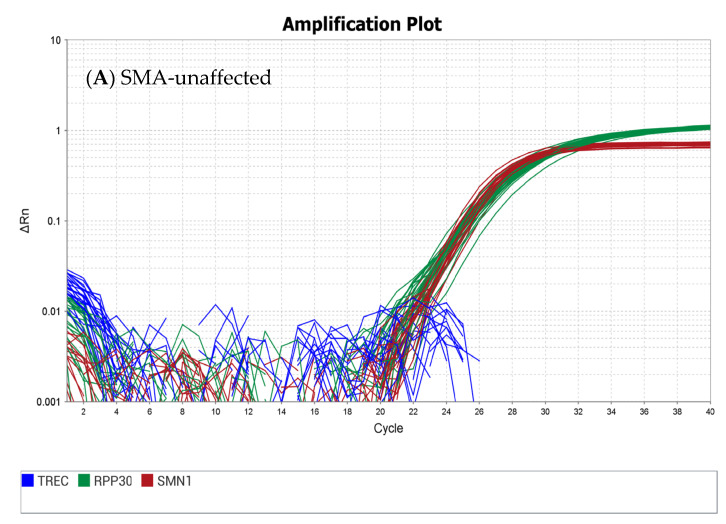

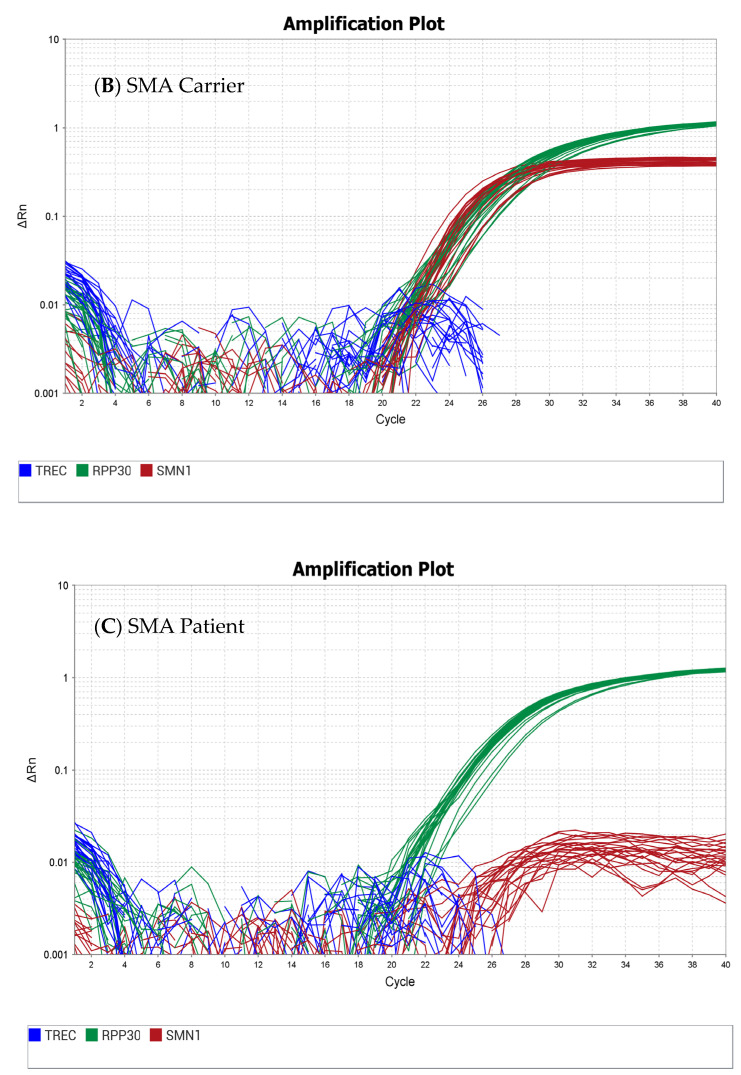
Real-time PCR amplification plots using the CDC assay from SMA quality assurance materials from cell lines established from de-identified donors with (**A**) normal *SMN1* exon 7 sequence, (**B**) heterozygous deletion of *SMN1* exon 7 sequence, and (**C**) homozygous deletion of *SMN1* exon 7 sequences.

Two SMA-positive (homozygous deletion of *SMN1* exon 7) cell lines were established, with two and three copies of *SMN2*, respectively. Additionally, a cell line with a single copy of *SMN1* exon 7 (carrier) and a negative control (two copies of the *SMN1* exon 7 sequence present) were established. All four cell lines were grown in large quantities, harvested, and added to leukocyte-depleted blood to create dried blood spots. The four sets of DBS materials were characterized using the CDC-developed method for *SMN1* exon 7, TREC, and the *RPP30* reference gene with the expected results (Figure 1). To ensure quality, CDC uses rigorous testing to validate that all materials are homogenous and fit for purpose.

Additionally, these materials have been used in routine proficiency testing and exceeded >80% consensus from all participants. This same process was used to create DBS samples that were distributed in the pilot external QC program on 21 May 2024. These DBSs can assist NBS labs in monitoring potential assay issues, including result variations between instruments, reagent lot-to-lot difference, false positive results caused by improper extraction or amplification, and false negative results caused by contamination.

### 3.3. Phase 3: Establishment of SMA PT Program

**Figure 2:**
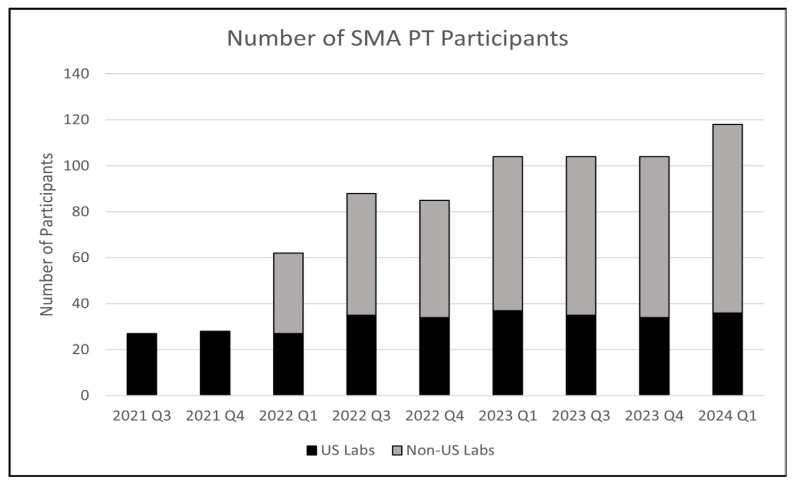
Number of participants in CDC’s Newborn Screening Quality Assurance Program, NSQAP, for SMA. Black bars represent U.S. participants, and grey represents the number of international participants.

A pilot PT program was successfully completed in 2020 with 28 U.S. NBS labs as participants. The program was transitioned to a routine program in 2021 and was then extended to include international labs in 2022 with 78 participants. As of 2024, 116 labs have enrolled in the SMA PT program (Figure 2).

As of Q1 2024, more than 73% of U.S. labs (25/34) used a laboratory-developed test (LDT), while a little more than two-thirds of the labs outside the U.S. (41/60) employed one of several commercial assay kits. Many of the participants utilized a multiplex assay that includes TREC and sometimes KREC (Table 1). Since a positive SMA screen is the absence of an *SMN1* amplification signal, it is important to ensure that the result is not due to insufficient DNA extracted from the sample or the presence of PCR inhibitors in the DNA. This is established by a robust amplification signal from an internal reference gene. There are a variety of reference genes in use. The most frequent reference genes used by NBS labs are an RNase P subunit, *RPP30*, or beta-actin. Other internal reference sequences being used include beta-globulin, H9, *SMN2* exon 8, *CFTR*, TREC, and chymotrypsin. Since the CDC quality assurance DBS materials are cell-based, they will cover equally well any and all internal reference genes that are part of the human genome. Also, most of the assays used to detect SMA were based on real-time PCR, although a high-resolution melting test and MALDI-TOF-based assays were included (Table 1).

**Figure 3:**
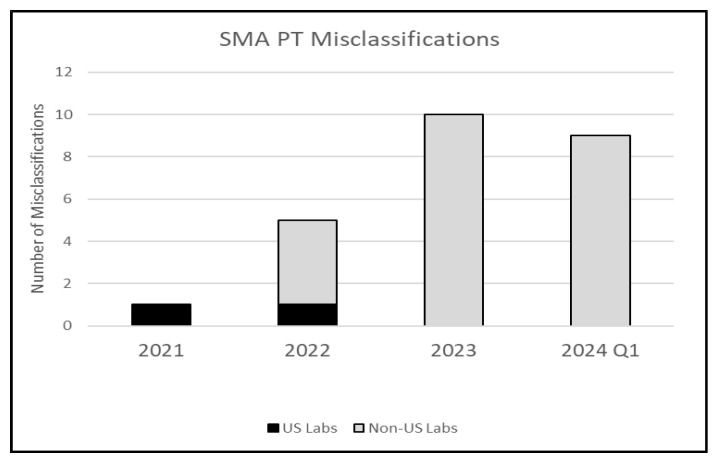
SMA PT misclassifications by year.

Since the inception of the SMA PT program over three years ago, the number of misclassified PT results has been relatively low. The number of misclassifications, which include both false positives and false negatives, over the course of a full year of results has been less than 2% (Figure 3 and Table 2).

### 3.4. Phase 4: External QC Program

Since most U.S. NBS labs currently use a laboratory-developed test to detect *SMN1* exon 7, external quality control materials are in high demand to monitor assays in routine day-to-day testing and to determine assay performance. CDC is in the process of initiating an external quality control program to provide certified QC materials to participating newborn screening labs. Participants will be asked to report their test results of the identified QC materials to a CDC NSQAP Participant Portal, after which individual results and de-identified analyses of all participants will be shared with individual labs so they can check how their assay compares to other NBS programs. Initially, the pilot program was distributed on 21 May 2024 to 47 U.S. and Canadian labs. Each laboratory received twenty 75 µL DBSs from each of three samples: an SMA-like/SCID-like (contains no *SMN1* exon 7 and no TREC), a normal sample with a TREC quantity near the population median, and a normal sample with a TREC quantity that is about 25% of the population median.

### 3.5. Ongoing CDC Technical Assistance

CDC has also provided technical assistance to the newborn screening community, including remote consultation and onsite support for assay development/implementation/validation, troubleshooting, and data analysis such as cutoff determination. Since 2021, we have fulfilled 183 requests related to SMA, including 54 requests from international labs located in 27 different countries and 134 requests from U.S. labs. The international requests came from 5 labs in Canada, 15 in Europe, 7 in Asia, 5 in Oceania, and 1 in South America. The most common request was for quality assurance materials, followed by technical advice and troubleshooting. To facilitate the transfer of knowledge, CDC scientists, in collaboration with the Association of Public Health Laboratories, host an annual hands-on training workshop where students are able to learn about and run the CDC-created SCID/SMA real-time PCR assay, among other techniques. Additionally, CDC scientists have served as contributors for the development of the Clinical Laboratory Standard Institute (CLSI) guidelines for SMA newborn screening that will soon be available [17].

## 4. Discussions

Newborn disorders included in the RUSP are chosen based on evidence that supports the potential net benefit of screening, the readiness and feasibility of states to screen for the disorder, and the availability of effective treatments [7]. As the leading cause of infant mortality due to a genetic disorder, SMA had been under observation by the newborn screening community for quite some time prior to its addition to the RUSP. The benefit to identifying affected infants early to prevent irreversible neuronal injury was consistent with the hallmarks of NBS disorders. Once effective therapies were available to treat SMA and arrest disease progression, a second key requirement was to ensure that an appropriate screening test was available. CDC’s Newborn Screening and Molecular Biology Branch laboratory proactively started developmental activities that would support NBS for SMA in 2013, having been alerted to the very promising results of the clinical trials of Spinraza^TM^. Phase I of CDC’s approach was to develop a molecular assay to detect the *SMN1* exon 7 deletion. To achieve this goal, CDC chose to use the real-time PCR assay platform for three reasons: the technology has a suitable throughput for the large sample volumes required in NBS, most U.S. NBS labs were already using similar technology to screen for SCID and thus had the necessary instrumentation and expertise, and the assay results were relatively easy to interpret. An added advantage of the real-time PCR assay was its potential to multiplex the SMA assay into the existing TREC assay for SCID, so that both could be detected in a single test. One of the challenges we faced was the inability of the test to discriminate between the *SMN1* gene and its highly similar paralog *SMN2*. We solved this challenge by adopting the use of LNA-modified probes. This approach has proven so successful that it was adopted by most U.S. NBS labs that use an LDT because it is easily multiplexed with the TREC assay already in use, has a fast turnaround time, and is easily adaptable for states with both high and low birth rates.

When SMA was added to the RUSP, the recommendation was for newborn screening laboratories to test for the homozygous deletion of exon 7 in the *SMN1* gene since this has been shown to account for >95% of SMA cases. The CDC-developed SMA assay enables labs to meet this requirement of identifying the presence or absence of *SMN1* exon 7. This assay, however, cannot detect newborns with SMA caused by other pathogenic variants such as a single nucleotide polymorphism (SNP) or small insertion/deletions (indel).

In Phase II, we developed quality assurance materials to be ready to support SMA screening. CDC is mandated by the U.S. Congress to provide DBS materials so NBS labs can monitor the analytical validity, utility, and performance of their assay. To fulfill this requirement, we developed cell-based DBS materials using de-identified patient cell lines established at CDC. Often, quality control materials are prepared using plasmids or synthetic DNA fragments. At CDC, we elected to produce cell-based DBSs because they more closely resemble real newborn samples, so that all aspects of the assay from DNA extraction through *SMN1* exon 7 detection can be evaluated. Additionally, cell-based materials contain the human genome and can be used for any assay platform, including genome or targeted sequencing, and contain any reference gene a lab selects. In Phase III, CDC used these cell-based QA DBSs to develop a PT program. The CDC Newborn Screening and Molecular Biology Branch was granted ISO/IEC 17043:2010 and 17034:2016 accreditation as a proficiency testing provider and producer of reference materials. The program, first initiated as a pilot in 2020, is now a routine program and serves 116 labs from around the world, including all U.S. NBS labs. We have seen a steady increase in enrollees from international labs during the last two years and are expecting the trend to continue. The final phase is Phase IV, which is almost completed, and the goal was to offer an external QC DBS program. The pilot QC program launched in May 2024.

As CDC has progressed through the different phases of SMA activities, we have consistently applied our increasing knowledge and capability to provide technical assistance to NBS labs that requested help in implementing SMA screening. We have shared our *SMN1* exon 7 assay design with many NBS labs, as well as with commercial assay manufacturers, to support the development of commercially available test kits. One frequent request from NBS labs has been for SMA DBS quality control materials, as labs using an LDT often cannot find a reliable source of QC materials in the DBS matrix. These materials are also useful in supporting labs using commercial test kits for the objective monitoring of the assay’s analytical performance. Once Phase IV, a combined TREC and SMA QC program, is fully implemented, this need will be met in a more systemic fashion. The CDC laboratory activities described in this manuscript illustrate a deliberate and logical path to effectively support newborn screening for SMA before and after its addition to the RUSP. CDC’s work in supporting U.S. public health laboratories to implement screening for SMA has facilitated nationwide screening as of February 2024, which is less than five years since its addition to the RUSP. This is the fastest national implementation in the U.S. of a newborn screening condition added to the RUSP since the inception of the RUSP. CDC’s process to support will serve as a road map for new disorders in the future.

## Figures and Tables

**Table 1:** Summary of all methods used by 109 NSQAP participants in the 2024 Quarter 1 PT event.

Method	Number of U.S. Laboratories	Number of Non-U.S. Laboratories	Targets	Total
LDT * Real-Time PCR–*SMN1*, TREC and reference gene run in a single tube	21	11	*SMN1*, TREC, Reference gene *	32
Revvity Eonis™ SCID-SMA kit	1	21	*SMN1*, TREC, *RPP30*	22
Revvity NeoMDx^TM^ RUO	9	3	*SMN1*, TREC, *RPP30*	12
ImmunoIVD SPOT-it™ TREC & *SMN1* Screening Kit or TREC, KREC, *SMN1* Kit	0	13	*SMN1*, TREC, beta-actin	13
LDT Real-Time PCR–*SMN1* and reference gene run in a single tube	3	2	*SMN1*, Reference gene **	5
ZenTech Targeted qPCR SMA	0	6	*SMN1, RPP30*	6
MRC Holland SALSA MC002 SMA Newborn Screen	0	2	*SMN1*	2
Trimaris SMA qPCR Tarama Kit	0	3	*SMN1, CFTR*	3
MALDI-TOF/MicroArray	0	2	*SMN1*, H9	2
Others	0	12	*SMN1*, Reference gene ***	12

* Reference gene (number of labs): *RPP30* (20), *RPPH1* (7), beta-actin (4), hemoglobulin subunit HPP (1). ** Reference gene (number of labs): *RPP30* (3), beta-actin (1), beta-globulin (1). *** Reference gene (number of labs): *RPP30* (3), beta-globulin (3), *RPPH1* (2), beta-actin (2), *SMN2* exon 8 (1), chymotrypsin (1).

**Table 2:** SMA PT misclassifications by year and by error type.

Year	Number of Reported Results	Number of Misclassifications	Error Type	
			False Positive	False Negative
2021	270	1 (0.3%)	0	1
2022	1055	5 (0.4%)	0	5
2023	1380	10 (0.7%)	4	6
2024Q1	545	9 (1.6%)	3	6

## Data Availability

No additional data.
